# Bicyclo­[2.2.1]hept-7-yl *p*-bromo­benzoate

**DOI:** 10.1107/S1600536812041268

**Published:** 2012-10-06

**Authors:** Barry A. Lloyd, Atta M. Arif

**Affiliations:** a Department of Chemistry, Weber State University, Ogden, Utah 84408-2503; b Department of Chemistry, University of Utah, Salt Lake City, Utah, 84112, USA

## Abstract

The title compound, C_14_H_15_BrO_2_, contains a sterically unencumbered norbornyl group. The dihedral angle between the plane of the carboxyl­ate group and the mean plane of the adjacent benzene ring is 5.3 (2)°. The dihedral angle between the plane of the carboxyl­ate group and the norbornyl methano C—O bond is 4.5 (1)°, the methano C atom deviating by 0.141 (2) Å from this plane. In the crystal, mol­ecules pack as pairs of enanti­omers, with a distance of 3.747 (1) Å between the centroids of nearest parallel benzene rings.

## Related literature

For calculated and experimental norbornane and related structures, see: Allinger *et al.* (1989 ▶); Pfund *et al.* (1980 ▶). For related polycyclic *p*-bromo­benzoate structures, see: Lloyd & Arif (2012 ▶); Lloyd *et al.* (1995 ▶, 2000 ▶). For a high resolution low temperature powder synchrotron X-ray diffraction structure of norbornane, see: Fitch & Jobic (1993 ▶). For some norbornyl bond lengths and angles, see: Watson *et al.* (1992 ▶). For possible C—O bond-length correlation to reactivity in a 7-norbornenyl benzoate, see: Jones *et al.* (1992 ▶).
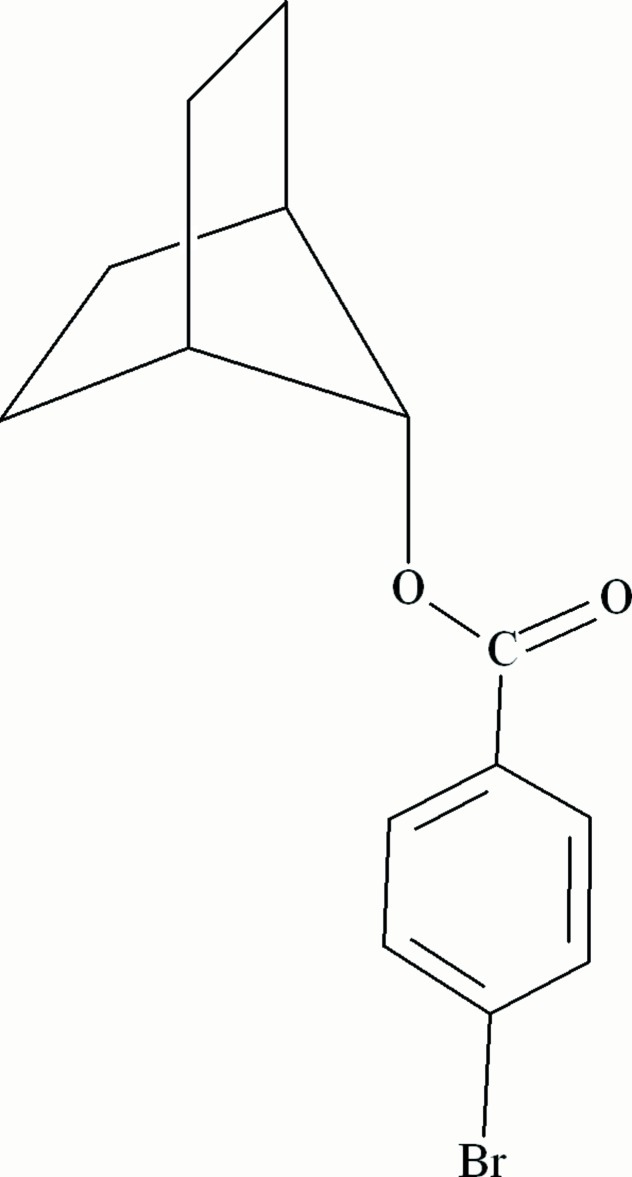


## Experimental

### 

#### Crystal data

C_14_H_15_BrO_2_
*M* *_r_* = 295.17
Monoclinic, 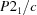

*a* = 11.7401 (2) Å
*b* = 6.3767 (1) Å
*c* = 17.7462 (3) Å
β = 109.584 (1)°
*V* = 1251.68 (4) Å^3^
*Z* = 4
Mo *K*α radiation
μ = 3.27 mm^−1^
*T* = 150 K
0.30 × 0.25 × 0.18 mm

#### Data collection

Nonius KappaCCD Diffractometer
Absorption correction: multi-scan (*DENZO-SMN*; Otwinowski & Minor, 1997 ▶) *T*_min_ = 0.440, *T*_max_ = 0.591
5495 measured reflections
2882 independent reflections
2469 reflections with *I* > 2σ(*I*)
*R*_int_ = 0.015

#### Refinement

*R*[*F* ^2^ > 2σ(*F* ^2^)] = 0.022
*wR*(*F* ^2^) = 0.055
*S* = 1.03
2882 reflections
215 parameters
All H-atom parameters refined
Δρ_max_ = 0.38 e Å^−3^
Δρ_min_ = −0.35 e Å^−3^

### 

Data collection: *COLLECT* (Nonius, 1998 ▶); cell refinement: *DENZO-SMN* (Otwinowski & Minor, 1997 ▶); data reduction: *DENZO-SMN*; program(s) used to solve structure: *SIR97* (Altomare *et al.*, 1999 ▶); program(s) used to refine structure: *SHELXL97* (Sheldrick, 2008 ▶); molecular graphics: *WinGX* (Farrugia, 2012 ▶), *ORTEP-3 for Windows* (Farrugia, 2012 ▶) and *PLATON* (Spek, 2009 ▶); software used to prepare material for publication: *Mercury* (Macrae *et al.*, 2008 ▶) and *publCIF* (Westrip, 2010 ▶).

## Supplementary Material

Crystal structure: contains datablock(s) I, global. DOI: 10.1107/S1600536812041268/fj2598sup1.cif

Structure factors: contains datablock(s) I. DOI: 10.1107/S1600536812041268/fj2598Isup2.hkl

Supplementary material file. DOI: 10.1107/S1600536812041268/fj2598Isup3.mol

Supplementary material file. DOI: 10.1107/S1600536812041268/fj2598Isup4.cml

Additional supplementary materials:  crystallographic information; 3D view; checkCIF report

## Figures and Tables

**Fig. 1.**
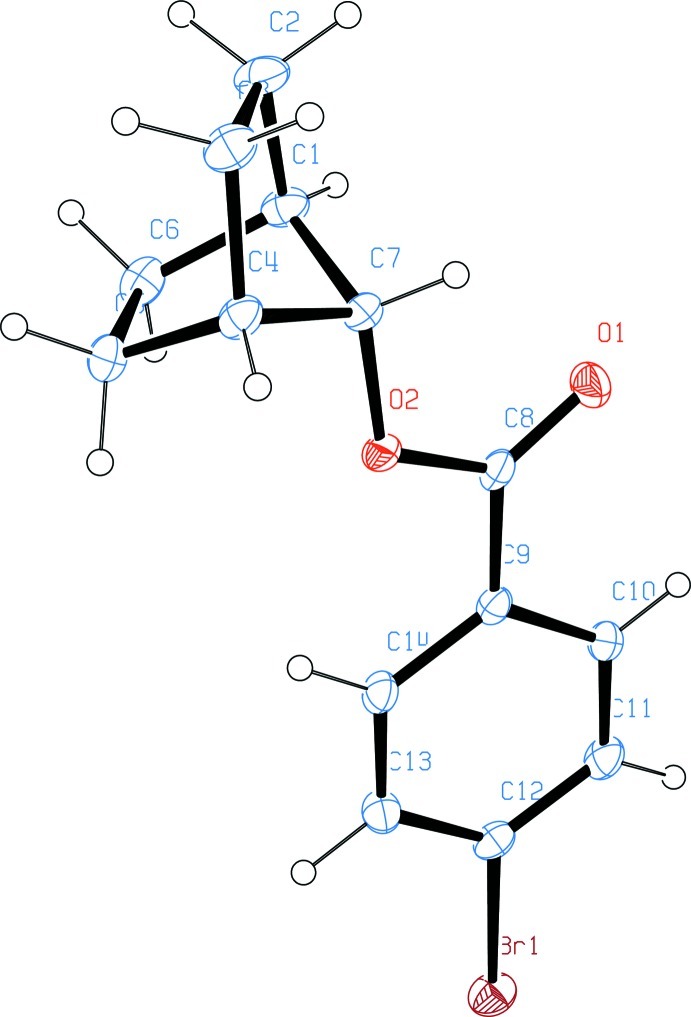
ORTEP-3 drawing of the title compound showing 50% displacement ellipsoids.

**Fig. 2.**
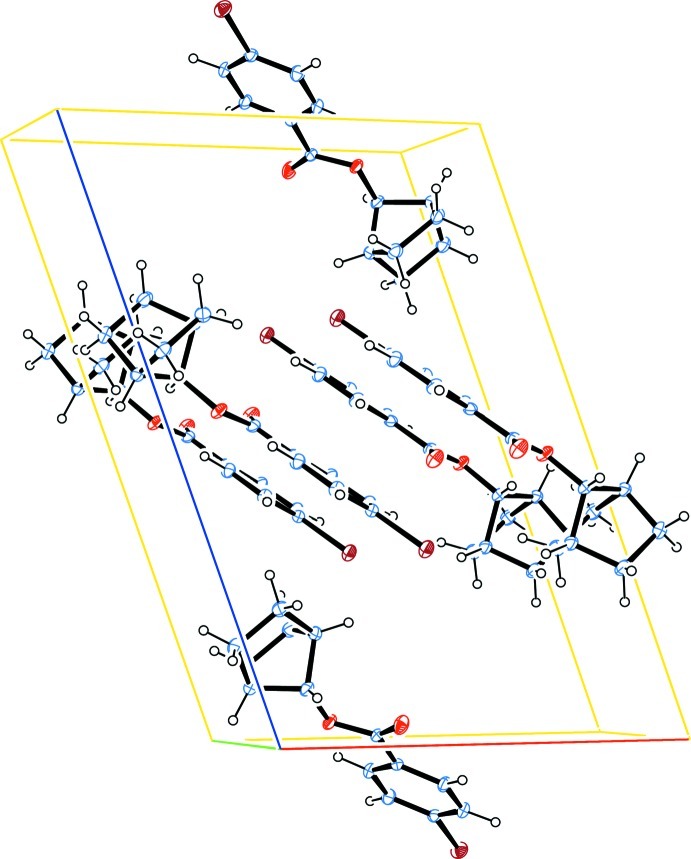
Cell packing diagram for the title compound.

**Fig. 3.**
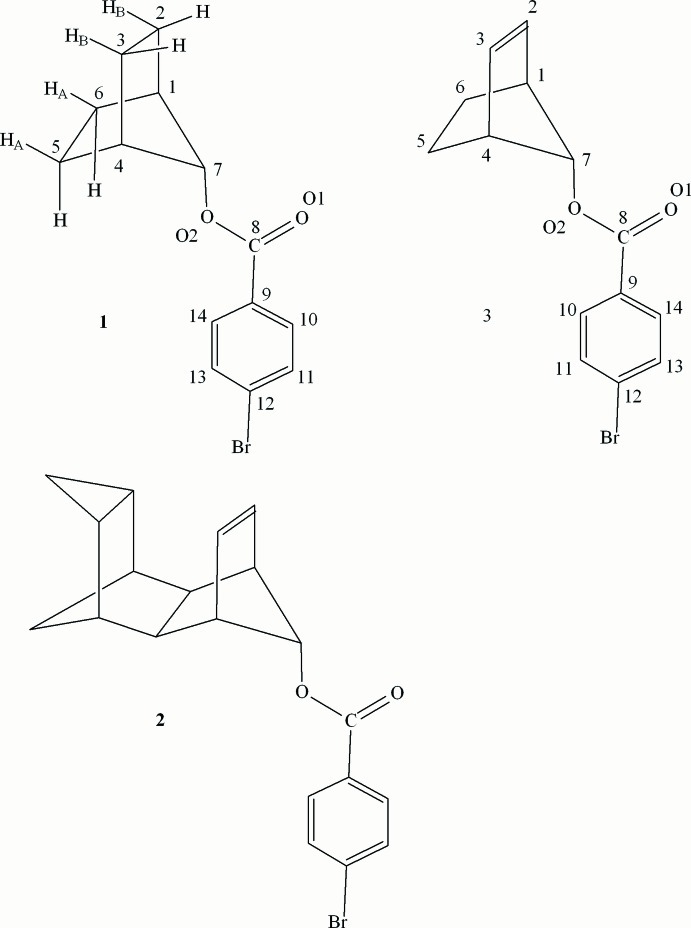
Compounds 1, 2, and 3.

## supplementary crystallographic information

### Comment

Many norbornyl structures have been previously determined, but precise bond lengths and angles are derivative dependent (Watson *et al.*, 1992). The Cambridge Crystallographic Database contains only five 7-norbornyl benzoate structures, but these are complicated by additional substituents and their associated steric interactions (for example, see Pfund *et al.*, 1980). An *ORTEP*-3 drawing (Farrugia, 2012) of title compound structure 1 is shown in Fig. 1, and a cell packing diagram is shown in Fig. 2. Structure 1 norbornyl group bond lengths and bond angles agree with reported values, but their precision is about ten times better than geometry averaged values (Watson *et al.*, 1992). Structure 1 was determined so that bond length, bond angle, least-squares plane, and nonbonding contact comparisons could be made with other related *p*-bromobenzoate structures (Lloyd & Arif, 2012, Lloyd *et al.*, 2000 and Lloyd *et al.*, 1995).

No nonhydrogen atom intermolecular contacts exist shorter than van der Waals radii sums, the smallest being O1^i^···C13^ii^ at 3.257 (2) Å [symmetry code: (ii) *x*, -1 + *y*, *z*]. Least squares planes are defined as C1—C7—C4 (plane 1), C1—C2—C3—C4 (plane 2), and C1—C6—C5—C4 (plane 3). Interplanar 1:2, 1:3, and 1:4 angles are: 121.2 (1)°, 125.5 (1)°, and 113.3 (1)°, respectively. Angle 1:2 is 1.4° larger than the corresponding 296 K structure 2 (Fig. 3) angle (Lloyd *et al.*, 1995), but smaller than corresponding angles of our other seven norbornenyl structures. Structure 1 angle 1: 3 is larger than analogous angles in all eight norbornenyl structures, and angle 2:3 is smaller for all except structure 3. Smaller 1:2 and larger 1:3 angles in structure 1 are likely a consequence of a longer C2—C3 norbornyl single bond (*versus* a shorter norbornenyl C2=C3 double bond) which bends C7 toward plane 2. The slightly larger structure 1 2:3 angle *versus*3 might result from intramolecular H3B···H5A and H2B···H6A contacts, 2.35 (3) and 2.40 (3) Å, respectively (Lloyd & Arif, 2012). Reactant structural features (such as C7—O2 bond length) that might portend the large norbornenyl: norbornyl solvolytic reactivity ratio are not obvious, and the late transition state idea (Jones *et al.*, 1992) is supported.

### Experimental

7-Norbornyl *p*-bromobenzoate (title compound 1) was made from commercial bicyclo[2.2.1]heptan-7-ol (7-norborneol, Alfa Products). Under a dry nitrogen atmosphere, 1.06 g freshly distilled (about 300 K, 7 Pa) *p*-bromobenzoyl chloride, 15 ml reagent grade dichloromethane, 0.802 g dry, freshly distilled (from CaH_2_ under N_2_) pyridine, and 0.540 g of sublimed (373 K, 7 Pa) 7-norborneol were combined and the mixture was refluxed for 15 min, then stirred for 2 d at 296 K. The reaction mixture was poured into 10 ml of 5% HCl solution, and layers were separated. The dichloromethane layer was washed twice more with 5% HCl solution, the dichloromethane was evaporated, and the residue was dissolved in about 3 ml of ether. The mixture was chromatographed on Florisil (petroleum ether, then ether). Recovered 1.22 g of 1, 85.3% yield, mp 350–351 K after two recrystallizations from petroleum ether/ether: ^1^H NMR (CDCl_3_, 90 MHz) δ 1.10–1.57 (4 H, m), 1.57–2.09 (4 H, m), 2.31 (2 H, m), 4.99 (1 H, s), 7.57 (2 H, d), 7.88 (2 H, d). Crystals were regrown slowly by dissolving 1.0 g of 1 in 5 ml of anhydrous ether in a 30 ml beaker. The beaker was placed inside a desiccator along with a 20 ml beaker containing 15 ml of petroleum ether (bp 303–333 K), and the desiccator was placed inside a freezer at 253 K. A one-hole rubber stopper was placed in the desiccator neck with glass wool inserted into the hole, allowing for slow evaporation. Crystals began forming after 3 d and they were filtered out after 5 d. One of these crystals was selected for X-ray analysis.

### Refinement

A colorless prism shaped crystal 0.30 × 0.25 × 0.18 mm in size was mounted on a glass fiber with traces of viscous oil and then transferred to a Nonius KappaCCD diffractometer equipped with Mo Kα radiation (λ = 0.71073 Å). Ten frames of data were collected at 150 (1) K with an oscillation range of 1 °/frame and an exposure time of 20 sec/frame (Nonius, 1998). Indexing and unit cell refinement based on all observed reflection from those ten frames, indicated a monoclinic *P* lattice. A total of 5495 reflections (Θ_max_ = 27.48°) were indexed, integrated and corrected for Lorentz, polarization and absorption effects using *DENZO– SMN* and *SCALEPAC* (Otwinowski & Minor, 1997). Post refinement of the unit cell gave a = 11.7401 (2) Å, b = 6.3767 (1) Å, c = 17.7462 (3) Å, β =109.584 (1)°, and V = 1251.68 (4) Å^3^. Axial photographs and systematic absences were consistent with the compound having crystallized in the monoclinic space group *P*2_1_/c.

The structure was solved by a combination of direct and heavy atom methods using *SIR*97 (Altomare *et al.*, 1999). All of the non-hydrogen atoms were refined with anisotropic displacement coefficients. Hydrogen atoms were located and refined isotropically using *SHELXL97* (Sheldrick, 2008). The weighting scheme employed was w = 1/[σ^2^(F_o_^2^) + (0.0254P)^2^ + 0.4883P] where P = (F_o_^2^ + 2F_c_^2^) /3. The refinement converged to R1 = 0.0224, wR2 = 0.0527, and S = 1.025 for 2469 reflections with I > 2σ(I), and R1 = 0.0294, wR2 = 0.0552, and S = 1.025 for 2882 unique reflections and 215 parameters, where R1 = Σ (|| F_o_ | – |F_c_ ||) / Σ |F_o_|, wR2 = [Σ(*w*(F_o_^2^ – F_c_^2^)2) / Σ(F_o_^2^)^2^]^1/2^, and S = Goodness-of-fit on F^2^ = [Σ (*w*(F_o_^2^ – F_c_^2^)^2^ / (n-p)] ^1/2^, n is the number of reflections and p is the number of parameters refined.

The maximum Δ/σ in the final cycle of the least-squares was 0.001, and the residual peaks on the final difference-Fourier map ranged from -0.35 to 0.382 e/Å^3^. Scattering factors were taken from the International Tables for Crystallography, Volume C, Chapters 4 pp 206–222 and 6 pp 476–516.

### Crystal data

**Table:**

C_14_H_15_BrO_2_	*F*(000) = 600
*M**_r_* = 295.17	*D*_x_ = 1.566 Mg m^−^^3^
Monoclinic, *P*2_1_/*c*	Melting point: 351 K
Hall symbol: -P 2ybc	Mo *K*α radiation, λ = 0.71073 Å
*a* = 11.7401 (2) Å	Cell parameters from 3135 reflections
*b* = 6.3767 (1) Å	θ = 1.0–27.5°
*c* = 17.7462 (3) Å	µ = 3.27 mm^−^^1^
β = 109.584 (1)°	*T* = 150 K
*V* = 1251.68 (4) Å^3^	Prism, colourless
*Z* = 4	0.30 × 0.25 × 0.18 mm

### Data collection

**Table:**

Nonius KappaCCD Diffractometer	2882 independent reflections
Radiation source: fine-focus sealed tube	2469 reflections with *I* > 2σ(*I*)
Graphite monochromator	*R*_int_ = 0.015
Phi and ω scans	θ_max_ = 27.5°, θ_min_ = 2.4°
Absorption correction: multi-scan (*DENZO-SMN*; Otwinowski & Minor, 1997)	*h* = −15→15
*T*_min_ = 0.440, *T*_max_ = 0.591	*k* = −8→8
5495 measured reflections	*l* = −22→23

### Refinement

**Table:**

Refinement on *F*^2^	Secondary atom site location: difference Fourier map
Least-squares matrix: full	Hydrogen site location: inferred from neighbouring sites
*R*[*F*^2^ > 2σ(*F*^2^)] = 0.022	All H-atom parameters refined
*wR*(*F*^2^) = 0.055	*w* = 1/[σ^2^(*F*_o_^2^) + (0.0254*P*)^2^ + 0.4883*P*] where *P* = (*F*_o_^2^ + 2*F*_c_^2^)/3
*S* = 1.03	(Δ/σ)_max_ = 0.001
2882 reflections	Δρ_max_ = 0.38 e Å^−^^3^
215 parameters	Δρ_min_ = −0.35 e Å^−^^3^
0 restraints	Extinction correction: *SHELXL*, Fc^*^=kFc[1+0.001xFc^2^λ^3^/sin(2θ)]^-1/4^
Primary atom site location: structure-invariant direct methods	Extinction coefficient: 0.0091 (7)

### Special details

**Table:**

Experimental. The program *DENZO-SMN* (Otwinowski & Minor, 1997) uses a scaling algorithm which effectively corrects for absorption effects. High redundancy data were used in the scaling program hence the 'multi-scan' code word was used. No transmission coefficients are available from the program (only scale factors for each frame). The scale factors in the experimental table are calculated from the 'size' command in the *SHELXL-97* input file.
Geometry. All e.s.d.'s (except the e.s.d. in the dihedral angle between two l.s. planes) are estimated using the full covariance matrix. The cell e.s.d.'s are taken into account individually in the estimation of e.s.d.'s in distances, angles and torsion angles; correlations between e.s.d.'s in cell parameters are only used when they are defined by crystal symmetry. An approximate (isotropic) treatment of cell e.s.d.'s is used for estimating e.s.d.'s involving l.s. planes.
Refinement. Refinement of *F*^2^ against ALL reflections. The weighted *R*-factor *wR* and goodness of fit *S* are based on *F*^2^, conventional *R*-factors *R* are based on *F*, with *F* set to zero for negative *F*^2^. The threshold expression of *F*^2^ > 2σ(*F*^2^) is used only for calculating *R*-factors(gt) *etc*. and is not relevant to the choice of reflections for refinement. *R*-factors based on *F*^2^ are statistically about twice as large as those based on *F*, and *R*- factors based on ALL data will be even larger.

### Fractional atomic coordinates and isotropic or equivalent isotropic displacement parameters (Å^2^)

**Table:**

	*x*	*y*	*z*	*U*_iso_*/*U*_eq_	
O1	0.29724 (11)	−0.03809 (19)	0.03440 (7)	0.0345 (3)	
O2	0.17768 (10)	0.23527 (18)	0.03757 (6)	0.0275 (2)	
Br1	0.470495 (15)	0.70360 (3)	−0.187891 (10)	0.03601 (8)	
C1	0.20441 (14)	0.1660 (3)	0.18033 (9)	0.0284 (3)	
C2	0.12314 (16)	0.0547 (3)	0.22003 (10)	0.0357 (4)	
C3	−0.00686 (16)	0.0964 (3)	0.16107 (11)	0.0341 (4)	
C4	0.01573 (14)	0.2271 (3)	0.09468 (9)	0.0268 (3)	
C5	0.05962 (15)	0.4456 (3)	0.12780 (10)	0.0313 (4)	
C6	0.18971 (16)	0.4036 (3)	0.18646 (10)	0.0336 (4)	
C7	0.13181 (14)	0.1242 (3)	0.09249 (9)	0.0253 (3)	
C8	0.26378 (13)	0.1393 (3)	0.01604 (8)	0.0244 (3)	
C9	0.31177 (13)	0.2786 (2)	−0.03379 (8)	0.0224 (3)	
C10	0.39565 (15)	0.1984 (3)	−0.06648 (9)	0.0274 (3)	
C11	0.44332 (15)	0.3225 (3)	−0.11205 (10)	0.0303 (4)	
C12	0.40660 (13)	0.5289 (3)	−0.12504 (8)	0.0257 (3)	
C13	0.32412 (15)	0.6139 (3)	−0.09307 (10)	0.0291 (3)	
C14	0.27768 (14)	0.4878 (3)	−0.04697 (9)	0.0270 (3)	
H1	0.2846 (17)	0.116 (3)	0.1972 (10)	0.029 (4)*	
H2A	0.1403 (19)	−0.093 (4)	0.2256 (12)	0.049 (6)*	
H2B	0.1354 (16)	0.109 (3)	0.2741 (11)	0.036 (5)*	
H3A	−0.0457 (17)	−0.037 (3)	0.1400 (12)	0.039 (5)*	
H3B	−0.0543 (18)	0.179 (3)	0.1874 (12)	0.038 (5)*	
H4	−0.0512 (17)	0.230 (3)	0.0436 (11)	0.031 (5)*	
H5A	0.0057 (17)	0.507 (3)	0.1529 (11)	0.034 (5)*	
H5B	0.0597 (17)	0.540 (3)	0.0856 (11)	0.035 (5)*	
H6A	0.2006 (18)	0.443 (3)	0.2418 (12)	0.046 (6)*	
H6B	0.2501 (18)	0.476 (3)	0.1699 (12)	0.043 (5)*	
H7	0.1235 (16)	−0.023 (3)	0.0794 (10)	0.029 (5)*	
H10	0.4167 (17)	0.061 (3)	−0.0576 (11)	0.038 (5)*	
H11	0.5023 (19)	0.270 (3)	−0.1319 (13)	0.042 (5)*	
H13	0.3001 (19)	0.760 (3)	−0.1028 (12)	0.040 (5)*	
H14	0.2213 (17)	0.543 (3)	−0.0241 (11)	0.035 (5)*	

### Atomic displacement parameters (Å^2^)

**Table:**

	*U*^11^	*U*^22^	*U*^33^	*U*^12^	*U*^13^	*U*^23^
O1	0.0420 (7)	0.0262 (6)	0.0433 (7)	0.0084 (5)	0.0250 (5)	0.0038 (5)
O2	0.0331 (6)	0.0299 (6)	0.0262 (5)	0.0085 (5)	0.0190 (5)	0.0049 (5)
Br1	0.03629 (11)	0.04188 (13)	0.03518 (11)	−0.00802 (7)	0.01902 (8)	0.00085 (8)
C1	0.0223 (7)	0.0396 (10)	0.0243 (7)	0.0034 (7)	0.0093 (6)	0.0054 (7)
C2	0.0356 (9)	0.0473 (11)	0.0288 (8)	0.0035 (8)	0.0169 (7)	0.0101 (8)
C3	0.0299 (8)	0.0427 (11)	0.0349 (9)	−0.0027 (8)	0.0177 (7)	0.0010 (8)
C4	0.0227 (7)	0.0357 (9)	0.0231 (7)	0.0029 (6)	0.0090 (6)	0.0000 (6)
C5	0.0347 (9)	0.0312 (9)	0.0333 (8)	0.0059 (7)	0.0184 (7)	−0.0013 (7)
C6	0.0326 (9)	0.0396 (10)	0.0301 (8)	−0.0049 (7)	0.0125 (7)	−0.0081 (8)
C7	0.0294 (8)	0.0267 (8)	0.0245 (7)	0.0020 (6)	0.0151 (6)	0.0015 (6)
C8	0.0244 (7)	0.0296 (8)	0.0203 (7)	0.0036 (6)	0.0090 (6)	−0.0045 (6)
C9	0.0214 (7)	0.0273 (8)	0.0179 (6)	0.0016 (6)	0.0056 (5)	−0.0030 (6)
C10	0.0304 (8)	0.0273 (8)	0.0285 (8)	0.0068 (7)	0.0153 (6)	0.0003 (7)
C11	0.0269 (8)	0.0389 (10)	0.0295 (8)	0.0053 (7)	0.0154 (6)	−0.0017 (7)
C12	0.0221 (7)	0.0343 (9)	0.0207 (7)	−0.0046 (6)	0.0074 (6)	−0.0025 (6)
C13	0.0300 (8)	0.0263 (8)	0.0326 (8)	0.0009 (7)	0.0125 (7)	−0.0010 (7)
C14	0.0266 (7)	0.0293 (8)	0.0284 (8)	0.0046 (6)	0.0134 (6)	−0.0027 (7)

### Geometric parameters (Å, º)

**Table:**

O1—C8	1.206 (2)	C5—C6	1.556 (2)
O2—C8	1.3421 (17)	C5—H5A	0.971 (19)
O2—C7	1.4469 (18)	C5—H5B	0.96 (2)
Br1—C12	1.8997 (15)	C6—H6A	0.98 (2)
C1—C7	1.529 (2)	C6—H6B	0.97 (2)
C1—C6	1.533 (3)	C7—H7	0.966 (19)
C1—C2	1.537 (2)	C8—C9	1.491 (2)
C1—H1	0.944 (18)	C9—C14	1.390 (2)
C2—C3	1.557 (2)	C9—C10	1.397 (2)
C2—H2A	0.96 (2)	C10—C11	1.377 (2)
C2—H2B	0.984 (19)	C10—H10	0.91 (2)
C3—C4	1.537 (2)	C11—C12	1.380 (2)
C3—H3A	0.98 (2)	C11—H11	0.94 (2)
C3—H3B	0.99 (2)	C12—C13	1.386 (2)
C4—C7	1.525 (2)	C13—C14	1.383 (2)
C4—C5	1.533 (2)	C13—H13	0.97 (2)
C4—H4	0.981 (19)	C14—H14	0.953 (19)
			
C8—O2—C7	117.02 (12)	C1—C6—H6A	110.0 (13)
C7—C1—C6	101.97 (13)	C5—C6—H6A	113.4 (12)
C7—C1—C2	99.62 (13)	C1—C6—H6B	109.8 (12)
C6—C1—C2	108.80 (14)	C5—C6—H6B	111.6 (12)
C7—C1—H1	114.8 (11)	H6A—C6—H6B	108.6 (17)
C6—C1—H1	116.0 (12)	O2—C7—C4	110.13 (13)
C2—C1—H1	113.8 (11)	O2—C7—C1	113.28 (13)
C1—C2—C3	103.42 (13)	C4—C7—C1	95.54 (12)
C1—C2—H2A	111.1 (13)	O2—C7—H7	110.2 (10)
C3—C2—H2A	111.2 (13)	C4—C7—H7	114.0 (11)
C1—C2—H2B	112.1 (12)	C1—C7—H7	113.0 (10)
C3—C2—H2B	112.7 (11)	O1—C8—O2	124.01 (14)
H2A—C2—H2B	106.5 (17)	O1—C8—C9	124.44 (13)
C4—C3—C2	103.05 (13)	O2—C8—C9	111.55 (13)
C4—C3—H3A	110.9 (11)	C14—C9—C10	119.00 (14)
C2—C3—H3A	109.6 (12)	C14—C9—C8	121.81 (13)
C4—C3—H3B	110.0 (11)	C10—C9—C8	119.17 (14)
C2—C3—H3B	110.7 (11)	C11—C10—C9	121.02 (15)
H3A—C3—H3B	112.2 (16)	C11—C10—H10	121.0 (12)
C7—C4—C5	102.27 (13)	C9—C10—H10	117.9 (12)
C7—C4—C3	99.88 (13)	C10—C11—C12	118.71 (14)
C5—C4—C3	108.73 (13)	C10—C11—H11	120.7 (13)
C7—C4—H4	115.4 (11)	C12—C11—H11	120.5 (13)
C5—C4—H4	113.6 (11)	C11—C12—C13	121.77 (15)
C3—C4—H4	115.4 (11)	C11—C12—Br1	119.64 (11)
C4—C5—C6	103.21 (13)	C13—C12—Br1	118.59 (13)
C4—C5—H5A	110.9 (11)	C14—C13—C12	118.87 (16)
C6—C5—H5A	114.0 (11)	C14—C13—H13	120.9 (12)
C4—C5—H5B	111.2 (11)	C12—C13—H13	120.2 (12)
C6—C5—H5B	111.8 (11)	C13—C14—C9	120.61 (14)
H5A—C5—H5B	105.9 (16)	C13—C14—H14	120.3 (12)
C1—C6—C5	103.36 (13)	C9—C14—H14	119.1 (12)
			
C7—C1—C2—C3	−35.57 (17)	C6—C1—C7—C4	−54.16 (14)
C6—C1—C2—C3	70.69 (17)	C2—C1—C7—C4	57.56 (15)
C1—C2—C3—C4	−0.06 (19)	C7—O2—C8—O1	6.3 (2)
C2—C3—C4—C7	35.81 (17)	C7—O2—C8—C9	−174.22 (12)
C2—C3—C4—C5	−70.86 (17)	O1—C8—C9—C14	−174.03 (15)
C7—C4—C5—C6	−33.78 (15)	O2—C8—C9—C14	6.5 (2)
C3—C4—C5—C6	71.24 (15)	O1—C8—C9—C10	4.3 (2)
C7—C1—C6—C5	34.25 (15)	O2—C8—C9—C10	−175.22 (13)
C2—C1—C6—C5	−70.38 (16)	C14—C9—C10—C11	−0.8 (2)
C4—C5—C6—C1	−0.36 (16)	C8—C9—C10—C11	−179.21 (15)
C8—O2—C7—C4	−166.14 (13)	C9—C10—C11—C12	0.0 (2)
C8—O2—C7—C1	88.22 (16)	C10—C11—C12—C13	0.5 (2)
C5—C4—C7—O2	−63.24 (15)	C10—C11—C12—Br1	−179.84 (12)
C3—C4—C7—O2	−175.05 (13)	C11—C12—C13—C14	0.0 (2)
C5—C4—C7—C1	54.04 (14)	Br1—C12—C13—C14	−179.71 (12)
C3—C4—C7—C1	−57.76 (15)	C12—C13—C14—C9	−0.9 (2)
C6—C1—C7—O2	60.55 (15)	C10—C9—C14—C13	1.3 (2)
C2—C1—C7—O2	172.27 (13)	C8—C9—C14—C13	179.63 (14)

## References

[bb1] Allinger, N. L., Geise, H. J., Pyckhout, W., Paquette, L. A. & Gallucci, J. C. (1989). *J. Am. Chem. Soc.* **111**, 1106–1114.

[bb2] Altomare, A., Burla, M. C., Camalli, M., Cascarano, G. L., Giacovazzo, C., Guagliardi, A., Moliterni, A. G. G., Polidori, G. & Spagna, R. (1999). *J. Appl. Cryst.* **32**, 115–119.

[bb3] Farrugia, L. J. (2012). *J. Appl. Cryst.* **45**, 849–854.

[bb4] Fitch, A. N. & Jobic, H. (1993). *J. Chem. Soc. Chem. Commun.* pp. 1516–1517.

[bb5] Jones, P. G., Kirby, A. J. & Percy, J. M. (1992). *Acta Cryst.* C**48**, 829–832.

[bb6] Lloyd, B. A. & Arif, A. M. (2012). *Acta Cryst.* E**68**, o2209. 10.1107/S1600536812027882 PMC3394005 22798870

[bb7] Lloyd, B. A., Arif, A. M. & Allred, E. L. (2000). *Acta Cryst.* C**56**, 1377–1379. 10.1107/s0108270100011185 11077305

[bb8] Lloyd, B. A., Arif, A. M., Coots, R. J. & Allred, E. L. (1995). *Acta Cryst.* C**51**, 2059–2062.

[bb9] Macrae, C. F., Bruno, I. J., Chisholm, J. A., Edgington, P. R., McCabe, P., Pidcock, E., Rodriguez-Monge, L., Taylor, R., van de Streek, J. & Wood, P. A. (2008). *J. Appl. Cryst.* **41**, 466–470.

[bb10] Nonius (1998). *COLLECT* Nonius BV, Delft, The Netherlands.

[bb11] Otwinowski, Z. & Minor, W. (1997). *Methods in Enzymology*, Vol. 276, *Macromolecular Crystallography*, Part A, edited by C. W. Carter Jr & R. M. Sweet, pp. 307–326. New York: Academic Press.

[bb12] Pfund, R. A., Schweizer, W. B. & Ganter, C. (1980). *Helv. Chim. Acta*, **63**, 674–681.

[bb13] Sheldrick, G. M. (2008). *Acta Cryst.* A**64**, 112–122. 10.1107/S0108767307043930 18156677

[bb14] Spek, A. L. (2009). *Acta Cryst.* D**65**, 148–155. 10.1107/S090744490804362X PMC2631630 19171970

[bb15] Watson, W. H., Kashyap, R. P., Krawiec, M., Marchand, A. P., Reddy, C. M. & Gadgil, V. R. (1992). *Acta Cryst.* B**48**, 731–737.

[bb16] Westrip, S. P. (2010). *J. Appl. Cryst.* **43**, 920–925.

